# Quantum mechanical MRI simulations: Solving the matrix dimension problem

**DOI:** 10.1126/sciadv.aaw8962

**Published:** 2019-07-19

**Authors:** Ahmed J. Allami, Maria Grazia Concilio, Pavan Lally, Ilya Kuprov

**Affiliations:** 1 School of of Medicine, University of Al-Ameed, Karbala, PO No: 198, Iraq.; 2 School of Chemistry, University of Southampton, Highfield, Southampton SO17 1BJ, UK.

## Abstract

We propose a solution to the matrix dimension problem in quantum mechanical simulations of MRI (magnetic resonance imaging) experiments on complex molecules. This problem is very old; it arises when Kronecker products of spin operators and spatial dynamics generators are taken—the resulting matrices are far too large for any current or future computer. However, spin and spatial operators individually have manageable dimensions, and we note here that the action by their Kronecker products on any vector may be computed without opening those products. This eliminates large matrices from the simulation process. MRI simulations for coupled spin systems of complex metabolites in three dimensions with diffusion, flow, chemical kinetics, and quantum mechanical treatment of spin relaxation are now possible. The methods described in this paper are implemented in versions 2.4 and later of the *Spinach* library.

## Teaser

The direct product between classical and quantum degrees of freedom may be left unopened.

## INTRODUCTION

There are two well-researched limits in the numerical simulation of magnetic resonance imaging: complicated spatial dynamics of simple spin systems and simple spatial dynamics of complicated spin systems. The former is diffusion and flow magnetic resonance imaging (MRI) of mostly water (*1*, *2*), and the latter is spatially encoded nuclear magnetic resonance (NMR) experiments (*3*, *4*) and localized NMR spectroscopy (*5*). Both cases are well covered by the existing simulation software. Both are also computationally straightforward because matrix dimensions are manageable: $\text{aut}({\mathbb{R}}^{3})⊗\text{su}(2)$ and ${\mathbb{R}}^{3}⊗\text{su}(2^{N})$ are both tractable, either directly or with reasonable approximations (*6*, *7*) for the matrix representations of $\text{su}(2^{N})$, where *N* is the number of spins.

The simulation problem becomes numerically intractable when complicated spatial dynamics (diffusion, convection, and flow in three dimensions) is combined with complicated spin dynamics (spin-spin coupling, cross-relaxation, chemical kinetics, etc.). A well-digitized three-dimensional (3D) phantom would have at least a hundred pixels in each of the three directions, meaning a dimension of at least 100^3^ = 10^6^ for the spatial dynamics generator matrices. At the same time, a typical metabolite (e.g., glucose) contains upwards of 10 coupled spins, meaning a Liouville space dimension of at least 4^10^ ≈ 10^6^. Direct products of spin and spatial dynamics generators would then have the dimension in excess of 10^12^ even before chemical kinetics is considered—clearly an impossible proposition even if sparse matrix arithmetic is used.

At the same time, sophisticated MRI and spatiotemporal NMR techniques are being increasingly called upon to report on neurotransmitters [e.g., acetylcholine (*8*) and γ-aminobutyric acid (GABA) (*9*)], bioenergetic metabolites [e.g., pyruvate (*10*) and lactate (*11*)], osmolytes [e.g., sarcosine (*12*) and taurine (*13*)], cellular membrane components [e.g., triglycerides (*14*) and cholesterol (*15*)], and other substances that, unlike water, feature nontrivial quantum mechanical spin processes in liquid phase. Metabolic imaging also implies the presence of chemical kinetics. Further away from the clinic, an increasing number of DNP (dynamic nuclear polarization) (*16*), PHIP (parahydrogen-induced polarization) (*17*), and singlet imaging proposals (*18*), and even patents (*19*), require accurate quantum mechanical treatment of spatially distributed spin dynamics, including simultaneous contributions from spin-spin coupling, cross-relaxation, convection, diffusion, flow, and chemical kinetics.

Numerical simulation of such systems used to be impossible in particular because the available software (*20*–*23*) always insisted on opening the Kronecker products in the general structure of the problem

[space dynamics]⊗[reaction kinetics]⊗[spin dynamics] (1)

when running the simulation. If all three matrices are large, then the resulting object has intractable dimensions. However, our long and careful look at the entire structure of the magnetic resonance simulation problem (*24*) produced an interesting observation: The only algebraic operation essentially required in any Liouville space magnetic resonance simulation is a matrix-vector product, where the matrix is a sum of objects with the structure shown in Eq. 1. Even time propagation, which technically involves a matrix exponential, can be reformulated using only matrix-vector operations (*25*), for example

$$\rho (t+\Delta t)=\text{exp}[-iL\Delta t]\rho (t)=\sum_{n=0}^{\infty }\frac{{\left(-i\Delta t\right)}^{n}}{n!}L(\ldots (L(\mathrm{L\rho})))$$ (2)

where **ρ** is the state vector, **L** is the Liouvillian superoperator, and *t* is time. The second important observation then comes from linear algebra—the action by a direct product on a vector may be computed without opening the direct product, for example

$$\left[A⊗B]v=\text{vec}[{\mathrm{BVA}}^{T}\right]$$ (3)

where **A** and **B** are matrices, **v** is a vector, **V** is obtained by reshaping **v** into appropriate dimensions for the product on the right-hand side, and vec stands for the column-wise stretch of a matrix back into a vector. The right-hand side of Eq. 3 is massively cheaper to compute. The entire class of such relations is well researched (*26*); they extend to multiple and nested direct products, as well as their sums.

We postulate here that direct products are best left unopened in magnetic resonance simulations, particularly in MRI, where the most problematic product is between spatial and spin degrees of freedom. This hypothesis is explored in this work: We report the design and implementation of a quantum mechanical MRI and spatiotemporal NMR simulation module in *Spinach* (*24*) that uses polyadics (the formal name for a sum of direct products) without opening them up. It is demonstrated below that previously unthinkable simulations now run in minutes.

## GENERAL SIMULATION FRAMEWORK

The MRI simulation problem contains three principal factors: (i) spatial distributions and spatial dynamics, including field maps of various coils, diffusion, and flow; (ii) chemical kinetics; and (iii) spin dynamics and relaxation. These factors are in a direct product relationship—each voxel may have different concentrations and transport velocities, each chemical species may have different spin dynamics, and spin dynamics may in turn be different in each voxel owing to, for example, a magnetic field gradient or a different viscosity that affects relaxation. Mathematically speaking, the structure of the problem is captured by Eq. 1 with the equation of motion consequently having the following algebraic form

$$\frac{d}{\mathrm{dt}}\rho (t)=[\sum_{\mathrm{nmk}}a_{\mathrm{nmk}}(t)M_{n}⊗K_{m}⊗S_{k}]\rho (t)$$ (4)

where **ρ**(*t*) is the state vector, *a_nmk_*(*t*) are interaction amplitudes, **M***_n_* are spatial operators, **K***_m_* are chemical kinetics operators (themselves possibly dependent on **ρ** if the kinetics is nonlinear), and **S***_k_* are (possibly dissipative) spin dynamics operators. For an infinitesimal time increment *dt*, the solution is

$$\rho (t+\mathrm{dt})=\text{exp}\{[\sum_{\mathrm{nmk}}a_{\mathrm{nmk}}(t)M_{n}⊗K_{m}⊗S_{k}]\mathrm{dt}\}\rho (t)$$ (5)

The problem of simulating each of the three compartments of Eq. 1 individually is comprehensively solved and extensively studied, from theoretical foundations (*27*–*29*) all the way to numerical implementations (*20*–*23*, *30*, *31*). However, the composite problem runs into the matrix dimension issue described in the Introduction. The solutions offered here revolve around compressing the dimensions of the three subproblems to the maximum possible extent and never opening the Kronecker products in Eq. 4. The insight that makes efficient simulation possible is empirical: The polyadic object appearing in Eq. 4 is always low rank. In other words, the sum is short and contains many unit matrices.

### Kronecker-times-vector operation

Consider the general case of a matrix-vector product where the matrix is a Kronecker product of smaller matrices, some of which may be unit matrices

$$y=[A^{\left(1\right)}⊗A^{\left(2\right)}⊗\ldots ⊗A^{\left(N\right)}]x$$ (6)

Each element of the object in square brackets is a product of the corresponding elements of **A**^(*k*)^

$${\left[\cdots \right]}_{i_{1}j_{1}i_{2}j_{2}\cdots i_{N}j_{N}}=a_{i_{1}j_{1}}^{\left(1\right)}a_{i_{2}j_{2}}^{\left(2\right)}\cdots a_{i_{N}j_{N}}^{\left(N\right)}$$ (7)

but calculating and storing the left-hand side is undesirable because the number of elements there is astronomical. It is more efficient to take the products on the right-hand side as they become necessary. In such a scenario, the multiplication count is unchanged, but the memory requirements would not exceed the resources already deployed in storing **A**^(*k*)^, all of which have reasonable dimensions.

The procedure for computing the product [⋯]**x** must then involve matching the linear index of the elements of **x** with the multi-index of $a_{i_{1}j_{1}}^{\left(1\right)}a_{i_{2}j_{2}}^{\left(2\right)}\cdots a_{i_{N}j_{N}}^{\left(N\right)}$. This is unexpectedly straightforward—**x** is reshaped into a multidimensional array whose dimensions match the row dimension of **A**^(*k*)^, each dimension is multiplied by the corresponding **A**^(*k*)^, and the result stretched back into a vector whose dimension is now the product of column dimensions of **A**^(*k*)^. This multiplication procedure, described by Fernandes *et al.* (*26*) and first implemented for *Matlab* by D. Gleich, proceeds as follows:

1) Record row dimensions of **A**^(*N*−*k*+1)^ into *c_k_*.
2) Reshape **x** into an *N*-dimensional array **X** with dimensions *c_k_*.
3) Loop index *n* over the dimensions of **X**.
Permute the dimensions of **X** to make its *n*th dimension leftmost.
Reshape **X** into a matrix of column dimension *c_n_* and row dimension $\prod_{m\neq n}c_{m}$.
Perform reassignment **X** = **A**^(*N*−*n*+1)^·**X** and replace *c_n_* by the column dimension of **A**^(*N*−*n*+1)^.
Reshape **X** back into an *N*-dimensional array **X** with dimensions *c_k_*.
Put the dimensions of **X** back into the original order.
4) Reshape **X** back into a vector and return it as **y**.

The extension of this procedure to a situation when **x** can have multiple columns is implemented in the *kronm* function supplied with *Spinach* versions 2.4 and later. Minor logistical optimizations are implemented: Multiplication by unit matrices is skipped, and memory-intensive dimension reordering operations are avoided for the first and the last instance of the loop.

### Polyadic object in *Spinach*

A considerable amount of software engineering is required before the method described in the previous section becomes useful for solving Eq. 4. The first substantial hurdle is addition: Eq. 4 is a sum of direct products; it cannot be written as a single direct product. However, because matrix-vector multiplication is distributive over addition, the algorithm reported in the previous section is easily extended to linear combinations of krons

(α[A⊗B⊗…]+β[C⊗D⊗…]+…)x=α[A⊗B⊗…]x+β[C⊗D⊗…]x+… (8)

In practice, this is implemented by buffering addition: When the user adds two Kronecker products, their sum is not evaluated—the terms are simply stored in a pointer array

A⊗B+C⊗D⊗E+… ⇔ {{A,B},{C,D,E},…} (9)

When the time comes to multiply this object into a vector, each term in the sum is multiplied into that vector individually, and the results are added up. We rely here on the fact that MRI evolution generators in Eq. 4 are short sums of Kronecker products: The number of terms is much smaller than the dimension of the matrix they represent. This also offers parallelization opportunities.

The second substantial hurdle is the norm—numerical implementations of Eq. 2 in finite precision arithmetic require the knowledge of the matrix norm. However, the immediate definition of a matrix norm requires either the largest singular value (2-norm), or sums across rows (infinity-norm) and columns (1-norm), or element-wise products (Frobenius norm). These are expensive operations. Thankfully, the Taylor series in Eq. 2 has infinite convergence radius, and therefore, only an estimate of the norm is actually required. The cheapest estimate we could find was published for 1-norm by Hager (*32*). It requires a few matrix-vector products, usually fewer than the subsequent evaluation of Eq. 2.

A less problematic but important observation is that the algorithm described in the previous section, particularly at the dimension reordering stages, has complicated memory access patterns. Nonsequential memory access can be expensive on modern central processing unit (CPU) architectures. The algorithm described in the previous section may therefore be viewed as a different trade-off between capacity and latency requirements on the random-access memory (RAM). However, the savings in the memory footprint are always large.

The last hurdle is that a polyadic object is sometimes pre-multiplied or post-multiplied by a small number of other objects. Because the only operation the entire object needs to deliver is the matrix-vector product, the best strategy is again to buffer the terms and apply them to the incoming vector before and after the polyadic is applied. The object structure is then extended as follows

$$\begin{array}{c} P_{1}\cdot \ldots \cdot P_{N}\cdot [A⊗B+C⊗D⊗E+\ldots ]\cdot Q_{1}\cdot \ldots \cdot Q_{M}\\ ⇕\\ \left\{P_{1},\ldots ,P_{N}\}\{\{A,B\},\{C,D,E\},\ldots \}\{Q_{1},\ldots ,Q_{M}\right\} \end{array}$$ (10)

The sequence is simply replayed from right to left every time a product into a vector is needed. Once the elements of this object are themselves allowed to be polyadic, the object can buffer an arbitrary sequence of additions, multiplications, and Kronecker products. This representation is only computationally efficient when the number of terms is much smaller than the dimension of the matrix it represents, but this is always the case for MRI evolution generators.

More sophisticated representations for tensor-structured objects have existed for some time (*33*, *34*). We do have a tensor train object in *Spinach* (*35*), but it is certainly not a simple machine-precision parameter-free black box of the same kind as polyadics. Because the polyadic decomposition in Eq. 4 is known a priori, and is nearly always already canonical, the benefits of going to advanced tensor-structured formats such as tensor trains (*33*) are not worth having to deal with their harrowing logistics.

A related design decision was to compress evolution generators into the polyadic format but to leave the state vector uncompressed. This is intentional and a result of much trial and failure—initially, we attempted to design a representation where both the generator and the state vector are compressed into a tensor structure (*33*, *34*) of some kind. However, none of the available tensor-structured formats were able to maintain sufficient accuracy in the state vector during time evolution simultaneously with sufficient extent of compression to justify their use. State vector compression is highly successful for iterative solvers returning stationary states of various kinds (*36*) but apparently not for long-range time evolution. In all magnetic resonance cases, we can reasonably foresee that the memory of modern computers is sufficient to leave the state vector uncompressed, and that is our recommendation.

### Synthetic benchmarks

Direct product component matrices for synthetic benchmarks were filled with normally distributed complex random numbers. The dimension of each matrix was chosen randomly between 1 and 64, and a random complex vector was generated to match the Kronecker product dimension. For sparse matrices, an optimistic nonzero density estimate was used: five complex nonzeroes per column. The matrix-vector multiplication operation was timed for 100 instances of this setup; the resulting statistics is presented in Table 1. The source code for this benchmark is available in versions 2.4 and later of the *Spinach* library (*24*).

**Table 1:** Wall clock time benchmarks for matrix-vector multiplication using a single Xeon E5-2698 CPU core in Matlab on a computer equipped with 256 GB of RAM.

**Matrix-vector multiplication task**	**Wall clock time, polyadic rep**	**Wall clock time, explicit rep**
[**A**⊗**B**]**v** with dim(**A**,**B**) ≤ 64, full	0.37 ± 0.01 ms	0.88 ± 0.12 ms
[**A**⊗**B**⊗**C**]**v** with dim(**A-C**) ≤ 64, full	1.8 ± 0.3 ms	Out of RAM
[**A**⊗**B**⊗**C**⊗**D**]**v** with dim(**A-D**) ≤ 64, full	97 ± 14 ms	Out of RAM
[**A**⊗**B**]**v** with dim(**A**,**B**) ≤ 64, sparse	0.21 ± 0.01 ms	0.05 ± 0.01 ms
[**A**⊗**B**⊗**C**]**v** with dim(**A-C**) ≤ 64, sparse	2.1 ± 0.3 ms	11.4 ± 1.6 ms
[**A**⊗**B**⊗**C**⊗**D**]**v** with dim(**A-D**) ≤ 64, sparse	105 ± 16 ms	Out of RAM

It is clear that 3D MRI experiments with quantum mechanical description of spin cannot be simulated on a system with 256 GB of RAM—this confirms the estimates given in the Introduction. However, multiplications using the polyadic object are all in milliseconds; they would remain realistic even if hundreds of terms are present in the sum in Eq. 4. There is no wall clock time advantage in these synthetic benchmarks, but the memory problem is solved. From the computational feasibility point of view, the polyadic object puts quantum mechanical 3D MRI simulations within reach.

### Matrix representations: Spatial dynamics generators

Given a concentration profile *c*(**r**, *t*), the diffusion flux is given by Fick’s first law (*37*), and the hydrodynamic flux is the product of concentration and flow velocity. The total flux therefore is

j(r,t)=v(r,t)c(r,t)−D(r,t)∇c(r,t) (11)

where ∇ = [∂/∂ ∂/∂y ∂/∂*z*]^T^ is the gradient operator, **v**(**r**, *t*) is the flow velocity field, and **D**(**r**, *t*) is the translational diffusion tensor field. We take both fields as given—spin processes in MRI are too weak to influence either diffusion or hydrodynamics. Any established solver (*38*, *39*) may therefore be used to obtain them before one starts the simulations covered here. Conservation of matter requires the local decrease in concentration to be equal to the divergence of its flux, and therefore

$$\frac{\partial }{\partial t}c(r,t)=-\text{div}[j(r,t)]=[\nabla^{T}\cdot v(r,t)+v^{T}(r,t)\cdot \nabla +\nabla^{T}\cdot D(r,t)\cdot \nabla ]c(r,t)$$ (12)

This is an instance of the Fokker-Planck equation with the probability density interpreted as concentration. A variety of other spatial dynamics models, covered in the literature dealing with the Fokker-Planck equation, may be used instead—rotational diffusion is a good example (*40*).

At the matrix representation level, finite difference matrices (*41*) suffice for the gradient operator; the question of numerical accuracy is explored in detail in the “Numerical accuracy conditions” section. The salient point here is that all differential operators appearing in Eq. 12 are direct products. For the gradient operator acting on the vectorized array of concentrations in every voxel

$$\nabla =[\begin{array}{c} \partial /\partial x\\ \partial /\partial y\\ \partial /\partial z \end{array}]\;\Rightarrow \;〚\nabla 〛=[\begin{array}{c} 〚\partial /\partial x〛⊗1_{Y}⊗1_{Z}\\ 1_{X}⊗〚\partial /\partial y〛⊗1_{Z}\\ 1_{X}⊗1_{Y}⊗〚\partial /\partial z〛 \end{array}]$$ (13)

where 〚*∂/∂x*〛 denotes a matrix representation of *∂/∂x* on a finite grid, and **1**_{X, Y, Z}_ are unit matrices of appropriate dimensions. If the diffusion tensor is constant and isotropic, then the generator has three terms

$$D(\frac{\partial^{2}}{\partial x^{2}}+\frac{\partial^{2}}{\partial y^{2}}+\frac{\partial^{2}}{\partial z^{2}})=D(〚\frac{\partial^{2}}{\partial x^{2}}〛⊗1_{Y}⊗1_{Z}+1_{X}⊗〚\frac{\partial^{2}}{\partial y^{2}}〛⊗1_{Z}+1_{X}⊗1_{Y}⊗〚\frac{\partial^{2}}{\partial z^{2}}〛)$$ (14)

where *D* is the diffusion coefficient. The dimension of each matrix in Eq. 14 is equal to the number of grid points along the corresponding dimension, typically of the order of 100. If the diffusion is anisotropic, then the number of direct product terms in the sum rises to nine. Even if the entire **D**(**r**, *t*) array depends so strongly on **r** that it must be stored explicitly on the finite grid, the direct product structure of Eq. 13 still only acts on one dimension at a time. The same applies to the flow generator.

Assuming hundreds of grid points in each of the three spatial directions, the biggest matrix dimension one would encounter in the polyadic form of Eq. 13, and consequently Eq. 12, is in the hundreds. The worst-case memory requirements would come from the velocity vector array and the diffusion tensor array: (hundreds)^3^ × (9 elements) × (64 bits per element) = about a gigabyte. This is well known to numerical hydrodynamics practitioners—the diffusion and flow problem in 3D is easily tractable. Finite difference derivative matrices are sparse (*41*).

### Matrix representations: Spin dynamics generators

The spin component of the equation of motion is

$$\begin{array}{c} \frac{\partial \rho (r,t)}{\partial t}=-iL(r,t)\rho (r,t)\\ L(r,t)=H(r,t)+iR(r) \end{array}$$ (15)

where **ρ**(**r**, *t*) is the spin density matrix at the spatial point **r** at time *t*, **H**(**r**, *t*) is the spin Hamiltonian commutation superoperator such that **Hρ** = **hρ** − **ρh**, and **R**(**r**) is the relaxation superoperator. The spin Hamiltonian contains chemical shift terms, pulsed field gradients, radiofrequency (RF) pulse terms, *J* couplings, and a great variety of other complications that are discussed in magnetic resonance textbooks (*28*)

$$\begin{array}{c} h(r,t)=-[B_{0}+B_{1Z}(r,t)+g^{T}(t)\cdot r]\sum_{n}(1+\delta_{n})\gamma_{n}S_{Z}^{\left(n\right)}-\\ -B_{1X}(r,t)\sum_{n}(1+\delta_{n})\gamma_{n}S_{X}^{\left(n\right)}-B_{1Y}(r,t)\sum_{n}(1+\delta_{n})\gamma_{n}S_{Y}^{\left(n\right)}-\\ +2\pi \sum_{n<k}J_{\mathrm{nk}}[S_{X}^{\left(n\right)}S_{X}^{\left(k\right)}+S_{Y}^{\left(n\right)}S_{Y}^{\left(k\right)}+S_{Z}^{\left(n\right)}S_{Z}^{\left(k\right)}]+\ldots \end{array}$$ (16)

where *B*_0_ is the primary magnet field (assumed to be directed along the *z* axis), **g** is the primary magnet field gradient vector, δ*_n_* are nuclear chemical shifts, γ*_n_* are nuclear magnetogyric ratios, $\left\{S_{X}^{\left(n\right)},S_{Y}^{\left(n\right)},S_{Z}^{\left(n\right)}\right\}$ are nuclear spin operators, *J_nk_* are internuclear scalar couplings (traditionally published in hertz, hence the 2π in front), *B*_1{X, Y, Z}_ are the Cartesian components of the RF magnetic field, etc.—Eq. 16 can be rather long, but for our purposes, **H**(**r**, *t*) is simply a well-understood matrix that standard software packages can return on demand. The same applies to the relaxation superoperator, which this communication is not a place to discuss.

An important point from the computational efficiency point of view is that the dimension of the Hilbert space spanned by a realistic spin system trajectory is usually much smaller than the dimension of the full Hilbert space of the spin system (*6*, *7*, *42*, *43*). A nonconstructive proof may be obtained from the fact that the dimension of the space spanned by the simulation trajectory

$$\left\{\rho_{0},{\mathrm{P\rho}}_{0},P^{2}\rho_{0},\ldots ,P^{N}\rho_{0}\},\;P=\text{exp}[-iL\Delta t\right]$$ (17)

is equal to the number of linearly independent vectors in it, which is smaller than or equal to the number of discrete time points in the trajectory (*42*). At a more physically motivated level:

1) Some spin states **σ** are not reachable from the initial condition under a given Hamiltonian and a given relaxation superoperator
  $$\langle \sigma |e^{-iLt}|\rho_{0}\rangle =0\;\forall t\in [0,t_{\text{sim}}]$$ (18)
  All states that cannot occur in the system trajectory may be dropped from the basis (*42*).
2) Some states are never populated to a significant extent because relaxation drains most of the amplitude en route. Any states that are reachable, but whose population stays below a set accuracy threshold, may also be dropped from the basis (*43*).
3) Some states are not reachable from the detection state. Any dynamics involving those states will never influence the observed parameter. For the purposes of simulating the dynamics of that parameter, such states may be dropped from the basis (*44*).

These observations are particularly relevant in liquid-state magnetic resonance, where the dimension of the space spanned by the system trajectory was recently shown to be orders of magnitude smaller than the dimension of the full Hilbert space (*45*).

Matrix representations of spin operators may be built directly in the reduced basis (*45*). The procedure makes use of the fact that the Lie algebra of operators acting on a multispin system is spanned by direct products of basis operators of the Lie algebras of individual spins

$$\overset{N}{\underset{k=1}{⊗}}\text{su}(2s_{k}+1)$$ (19)

where *N* is the number of spins and *s_k_* is the quantum number of each spin. The only consistent way to remove a specific generator **O***_m_* from a Lie algebra while preserving the rest of its structure is to modify the corresponding structure coefficients

$$[O_{i},O_{j}]=\sum_{k}c_{\mathrm{ijk}}O_{k}$$ (20)

by zeroing the coefficients *c_ijm_* that lead into the “ignored” physical states. The resulting object is still a Lie algebra that now generates the time dynamics of the approximate physical model under the exponential map. The structure coefficients of the direct product algebra are related to the structure coefficients of the single-spin algebras in a computationally friendly way because

$$\begin{array}{cc} \text{Tr}(O_{i}O_{j}O_{k}^{†}) & =\text{Tr}[(\overset{N}{\underset{n=1}{⊗}}S_{\text{in}})(\overset{N}{\underset{n=1}{⊗}}S_{\mathrm{jn}}){\left(\overset{N}{\underset{n=1}{⊗}}S_{\mathrm{kn}}\right)}^{†}]\\ & =\text{Tr}[\overset{N}{\underset{n=1}{⊗}}(S_{\text{in}}S_{\mathrm{jn}}S_{\mathrm{kn}}^{†})]=\prod_{n=1}^{N}\text{Tr}(S_{\text{in}}S_{\mathrm{jn}}S_{\mathrm{kn}}^{†}) \end{array}$$ (21)

where **S***_kn_* is the *k*th generator of the Lie algebra of the *n*th spin—usually a 2 × 2 matrix. The cost of computing individual structure coefficients of the full spin system algebra is thus linear with respect to the total number of spins. Once the structure coefficients are available, matrix representations may be built in any basis—see (*45*) for further information. When the restricted basis scales polynomially with the size of the spin system [true in liquid state (*43*)], this yields an overall polynomially scaling simulation algorithm. For our purposes here, all relevant methods are published (*6*, *42*–*47*) and implemented in *Spinach* (*24*). The program simply provides the matrices on demand.

### Matrix representations: Spin-space coupling

All interaction operators, initial states, and detection states that couple spin and spatial degrees of freedom are also short polyadic sums. The initial spin state need not be the same across the sample, but the number of initial spin states that realistically occur in magnetic resonance simulations is small, meaning that the corresponding polyadic sum

$$\rho =\sum_{k}\Phi_{k}^{\left(\rho \right)}⊗\rho_{k}$$ (22)

is short. In this equation, the sum runs over all initial spin states **ρ***_k_* that occur in the sample, and $\Phi_{k}^{\left(\rho \right)}$ are the corresponding “phantoms”—3D arrays that give the amplitude of **ρ***_k_* at each point of the sample, including variations that pertain to concentrations of individual substances. The same argument applies to the detection state, where the role of the phantoms is played by the coil receptivity profiles **Φ**_{X, Y, Z}_

$$\sigma =\Phi_{X}⊗\sum_{n}(1+\delta_{n})\gamma_{n}S_{X}^{\left(n\right)}+\Phi_{Y}⊗\sum_{n}(1+\delta_{n})\gamma_{n}S_{Y}^{\left(n\right)}+\Phi_{Z}⊗\sum_{n}(1+\delta_{n})\gamma_{n}S_{Z}^{\left(n\right)}$$ (23)

On the operator side, location dependence is important in the relaxation superoperator because it is a source of contrast in MRI. Another polyadic decomposition makes an appearance, this time with a sum over different relaxation mechanisms

$$R=\sum_{k}\Phi_{k}^{\left(R\right)}⊗R_{k}$$ (24)

where **R***_k_* are the relaxation superoperators responsible for the individual relaxation mechanisms and $\Phi_{k}^{\left(R\right)}$ are the corresponding “phantoms”—cubes of data describing the amplitude of each relaxation mechanism at each voxel in the sample. It is an experimental observation that this sum is short.

The coherent spin Hamiltonian need not be the same in every voxel either. The matrix representation of the pulsed field gradient part of the Hamiltonian is again a short polyadic sum

$$H_{\text{PFG}}(t)=-[\begin{array}{c} g_{X}(t)X⊗1_{Y}⊗1_{Z}+\\ g_{Y}(t)1_{X}⊗Y⊗1_{Z}+\\ g_{Z}(t)1_{X}⊗1_{Y}⊗Z \end{array}]⊗\sum_{n}(1+\delta_{n})\gamma_{n}S_{Z}^{\left(n\right)}$$ (25)

where {**X**, **Y**, **Z**} are matrices containing grid point coordinates on the diagonal, and {**1**_X_, **1**_Y_, **1**_Z_} are unit matrices of appropriate dimensions. The expression in the square brackets is easily extended, by adding terms such as **X**^2^ ⊗ **Y** ⊗ **1**_Z_, to account for the non-uniformity of the gradients produced by realistic gradient coils.

The RF Hamiltonian depends on spatial coordinates because the magnetic field produced by the RF coils is not uniform either. It has exactly the same structure as Eq. 23, only with time-dependent amplitude coefficients in front

$$\begin{array}{cc} H_{\text{RF}}(t)= & a_{X}(t)\Phi_{X}⊗\sum_{n}(1+\delta_{n})\gamma_{n}S_{X}^{\left(n\right)}+\\ & +a_{Y}(t)\Phi_{Y}⊗\sum_{n}(1+\delta_{n})\gamma_{n}S_{Y}^{\left(n\right)}+\\ & +a_{Z}(t)\Phi_{Z}⊗\sum_{n}(1+\delta_{n})\gamma_{n}S_{Z}^{\left(n\right)} \end{array}$$ (26)

All matrices mentioned in Eqs. 22 to 26 can have their own direct product structure—nested polyadics are supported by the object described in the “Polyadic object in *Spinach*” section, where individual arrays may be polyadics themselves. For reasons of brevity, we did not mention less common spin-space couplings here, but it stands to reason that all of them are short sums of Kronecker products. Generalizations may be made into solid-state NMR, EPR (electron paramagnetic resonance), and other types of magnetic resonance spectroscopy. The algebra here is actually obvious and well known to specialists in this field—the challenge is rather in making full use of this direct product structure. It is also in the software engineering, where the problem of implementing all of the above in a user-friendly, flexible, and general way is formidable.

### Matrix representations: Chemical kinetics

A comprehensive treatment of chemical kinetics in the general magnetic resonance context was given by Kühne *et al.* (*48*) and discussed by Ernst *et al.* [eq. 2.4.34 and fig. 2.4.2 in (*49*)]. The equation of motion for the concatenated state vector **ρ**^c^ of all chemical subsystems is

$$\frac{d\rho^{c}}{\mathrm{dt}}=[-iH^{c}(t)+R^{c}+K^{c}(t)]\rho^{c}$$ (27)

where **H**^c^(*t*) is a diagonal concatenation of the Hamiltonian commutation superoperators, **R**^c^ is a similar concatenation of thermalized relaxation superoperators, and **K**^c^(*t*) is a pseudolinear superoperator that depends on all concentrations and reaction rates but still has a tensor structure—it is a sum of Kronecker products between chemical reaction operators and spin projection operators, all Kroneckered with whatever other degrees of freedom there may be.

The general case is notable for its Daedalean notation (*48*), but the principle is easy to illustrate using an exchange process between two conformations A and B

$$\frac{d}{\mathrm{dt}}[\begin{array}{c} \rho_{A}\\ \rho_{B} \end{array}]=[-i(\begin{array}{cc} H_{A} & 0\\ 0 & H_{B} \end{array})+(\begin{array}{cc} R_{A} & 0\\ 0 & R_{B} \end{array})+(\begin{array}{cc} -k_{+}1 & +k_{-}1\\ +k_{+}1 & -k_{-}1 \end{array})][\begin{array}{c} \rho_{A}\\ \rho_{B} \end{array}]$$ (28)

where the block structure is directly visible, and the savings obtained by respecting it are obvious.

Because chemical exchange between nodes of a grid converges to diffusion as the grid becomes finer, the same mathematics also covers stochastic Liouville equation formulation (*50*) of relaxation theory. Cyclic networks of unidirectional processes converge to periodic motions such as magic angle spinning (*51*).

## NUMERICAL ACCURACY CONDITIONS

Diffusion and flow operators on finite grids have two popular matrix representations: finite difference matrices (*41*) and spectral differentiation matrices (*52*). The accuracy of the former depends on the grid spacing and the choice of the finite difference coefficients; both methods require all spatial frequencies to be below the Nyquist frequency of the grid.

On the Nyquist condition side, for the expected cases of laminar flow and diffusion, the dominant source of high frequencies along spatial coordinates are pulsed field gradients. An accurate simulation is the one that guarantees correct treatment of diffusive and hydrodynamic transport for the tightest phase spiral that the pulse sequence can generate in any element of the spin density matrix. Because this includes unobservable and observable coherences, the upper bound on the highest spatial frequency Ω_max_ must assume that each gradient, even if intended to refocus some spin states, would continue to defocus some other states. Therefore, in each spatial direction

$$\Omega_{\text{max}}\leq \frac{L}{2B_{0}}{\left\|H_{Z}\right\|}_{2}\int |g(t)|\mathrm{dt}$$ (29)

where *L* is the length of the sample in the chosen dimension, pulsed field gradients are assumed to be zero in the middle, *B*_0_ is the magnet induction, ‖**H**_Z_‖_2_ is the 2-norm (largest absolute eigenvalue) of the Zeeman Hamiltonian, *g*(*t*) is the gradient amplitude in the chosen dimension as a function of time, and the integral is taken over the duration of the experiment. The grid point spacing *h* is limited by the Nyquist condition (*53*) to have more than two grid points per period of Ω_max_

$$\Omega_{\text{max}}h<\pi$$ (30)

For sequences that make extensive use of gradients, the bound in Eq. 29 may be overly cautious. A simple practical test is to perform spatial Fourier transforms after each gradient event in the pulse sequence and to inspect the edges of the frequency domain representation. Nonzero amplitude at the edges would indicate that the number of points in the spatial grid must be increased.

Equation 30 is the only accuracy condition when Fourier spectral differentiation matrices are used. However, they are expensive because they are not sparse. For diffusion and flow, it is therefore common to use finite difference matrices instead; they have further accuracy conditions on the grid point spacing. These conditions follow from Taylor series expressions for derivatives on finite grids. For the simplest example of the central first derivative

$$\begin{array}{c} f(x+h)=f(x)+f\prime (x)h+\frac{f^{\prime \prime }(x)}{2}h^{2}+\int_{x}^{x+h}f\prime \prime \prime (t)\frac{{\left(x+h-t\right)}^{2}}{2}\mathrm{dt}\\ f(x-h)=f(x)-f\prime (x)h+\frac{f^{\prime \prime }(x)}{2}h^{2}+\int_{x}^{x-h}f\prime \prime \prime (t)\frac{{\left(x-h-t\right)}^{2}}{2}\mathrm{dt} \end{array}$$ (31)

where Lagrange’s remainders (*54*) are used after second order. Solving this for *f*′(*x*) yields

$$f\prime (x)=\frac{f(x+h)-f(x-h)}{2h}+R(x,h)$$ (32)

The functions being differentiated are dominated by gradient spirals with the worst-case frequency obtained in Eq. 29. The worst case is therefore *f*(*x*) = exp(− *i*Ω_max_*x*). This permits a more detailed examination of the remainder *R*(*x*, *h*) in Eq. 32. After taking the integrals and simplifying the result, we obtain the following expression for the relative error when ∣Ω_max_*h*∣ < 1

∣R(x,h)f′(x)∣=1−sin(Ωhmax)Ωhmax≈(Ωhmax)26≪1 (33)

This condition is more stringent than Eq. 30 and necessitates a finer grid; this is the price to pay for the sparsity of the differentiation operators.

A variety of other finite difference schemes are available in the literature (*41*), usually accompanied by the accuracy estimate of the form *O*[*h^n^*], where *h* is the grid spacing and *n* is a small integer. By following the same procedure as the one in Eqs. 31 to 33, it is easy to obtain a more accurate estimate of the worst-case relative error on the derivative. In our context, it is *O*[(Ω _max_ *h*)*^n^*], where Ω _max_ is the maximum spatial frequency that occurs in the experiment. In practice, it is reasonable to start from ∣Ω _max_ *h*∣ ≪ 1 and to make the grid finer until the simulation result no longer changes.

**Fig. 1:**
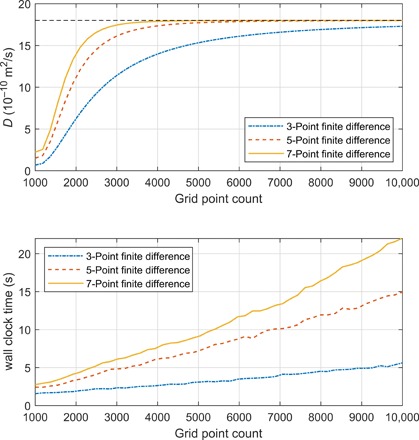
Accuracy and run time statistics for a Stejskal-Tanner pulse sequence simulation followed by extraction of the known diffusion coefficient (18 × 10^−10^m^2^/s, dashed line) from the simulated data. The 1.5-cm-long sample contains a single type of protons with a chemical shift of 4.6 ppm at 11.74 T. The Stejskal-Tanner pulse sequence simulation uses ideal RF pulses; perfectly rectangular gradients are assumed with no stabilization delay. The duration of gradient pulses is δ = 2 ms, and the duration of the diffusion delay is Δ = 50 ms. Gradient amplitudes varied from 0 to 0.5 T/m. (Top) The diffusion coefficient extracted by fitting Eq. 34 to the simulated data for three finite difference stencil sizes as a function of the spatial grid size. (Bottom) Wall clock time (2 × Intel Xeon E5-2698) for the pulse sequence simulation for three finite difference stencil sizes as a function of the spatial grid size.

A good practical example of this is the ideal Stejskal-Tanner experiment (*55*), for which the analytical expression for the signal attenuation due to isotropic diffusion is known

$$S(g)=S(0)e^{-\gamma^{2}\delta^{2}g^{2}D(\Delta -\delta /3)}$$ (34)

where *S*(*g*) is the signal intensity in an experiment with the gradient amplitude *g*, γ is the magnetogyric ratio (including any applicable chemical shift corrections) of the working spins, δ is the duration of the gradient pulse, Δ is the duration of the diffusion delay, and *D* is the diffusion coefficient. Running a numerical Stejskal-Tanner simulation on a finite grid and then fitting Eq. 34 to extract the diffusion coefficient back provides an illustration of how numerical accuracy depends on the grid spacing and the finite difference stencil size (Fig. 1).

Figure 1 quantitatively illustrates the problem described in the Introduction: Accurate simulations of diffusion NMR experiments require thousands of points in each spatial dimension. Even with sparse matrix arithmetic, 3D samples with complicated spin systems and chemical processes are beyond modern computers unless Kronecker products are left unopened in Eq. 4. The minimum grid satisfying the Nyquist condition for spatial frequencies in Fig. 1 has 1280 points; Fourier spectral derivative operators become accurate from that size onward. However, the wall clock time is actually much longer with a 2000-point Fourier differentiation operator than with a 10,000-point finite difference operator because Fourier operators are not sparse.

**Fig. 2:**
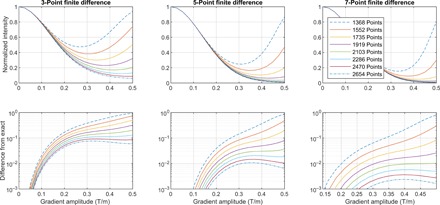
Simulated Stejskal-Tanner diffusion attenuation profiles as a function of spatial grid size. The 1.5-cm-long sample contains a single type of protons with a chemical shift of 4.6 ppm at 11.74 T. The Stejskal-Tanner pulse sequence simulation uses ideal RF pulses; perfectly rectangular gradients are assumed with no stabilization delay. The duration of gradient pulses is δ = 2 ms, and the duration of the diffusion delay is Δ = 50 ms. The diffusion coefficient is 18 × 10^−10^ m^2^/s. (Top) Diffusion attenuation profiles for spatial grids and finite difference stencils of different sizes (the minimal grid that satisfies the spatial Nyquist condition in this system has 1280 points). (Bottom) Difference between the simulated diffusion attenuation profiles and the exact analytical answer for grids and finite difference stencils of difference sizes.

The same accuracy analysis may be viewed from a different perspective: as a condition on the maximum accumulated gradient winding that a given spatial grid can support. This is illustrated in Fig. 2, which shows a comparison between signal attenuation observed in the simulation and the exact analytical solution as a function of the grid size and the differentiation stencil.

The simulation monotonically becomes more accurate as the grid point count increases. The practical conclusion again is that it is reasonable to use a seven-point stencil, to start from about twice the point count dictated by the Nyquist condition, and to increase the point count until the simulation result stops changing to the accuracy required. The exponential convergence illustrated in the bottom row of Fig. 2 stands in sharp contrast with the linear convergence of Monte Carlo methods (*56*), which are the only viable alternative in situations when quantum mechanical spin dynamics coexists with spatial motion. Even if Monte Carlo MRI simulation engines using the Liouville–von Neumann equation in the spin subspace existed (they currently do not), a grid solver would always converge faster (*57*).

**Fig. 3:**
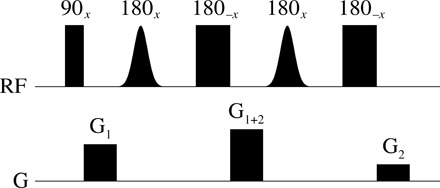
DPFGSE pulse sequence element (*67*) setup to defocus the signals influenced by the pair of soft pulses. In our experience, this is the best solvent suppression method in existence. The sequence may be switched from selective suppression to selective excitation by turning off the hard 180° pulses.

An important consideration is the type of the boundary condition. Fourier differentiation matrices require periodic boundaries, and finite difference operators for diffusion and flow all have a particularly simple form in periodic boundary conditions. The logistics of implementing other types of boundaries in a general simulation package is formidable, and therefore only the periodic boundaries are supported in version 2.4 of *Spinach* (*24*). In practice, this means that sufficient white space must be left on either side of the sample concentration phantom to prevent diffusion and flow processes from folding over.

## NUMERICAL EFFICIENCY

### Computationally efficient time propagation

The methods listed in the “Matrix representations: Spin dynamics generators” section can be applied a priori to decide which spin states would, in practice, contribute to the dynamics. However, when the equations of motion are integrated in the resulting matrix-vector representation, there are further efficiency opportunities—problem dimension may be reduced further by looking at the actual trajectory. These “trajectory level” methods include the following:

1) Krylov subspace analysis: All simulation trajectories have finite duration and finite step length. The space spanned by the trajectory vectors is known as the Krylov subspace of the propagator matrix (*25*). The dimension of that subspace (i.e., the number of linearly independent vectors in its basis) in favorable cases can be orders of magnitude smaller than the full state space dimension (*42*).
2) Zero track elimination: If a particular state has remained unpopulated within a certain initial period in the system evolution, rigorous bounds can be placed on its subsequent contribution to the system dynamics (*42*). States that remain unpopulated up to a user-specified tolerance can be dropped.
3) Liouvillian path tracing: Spin dynamics generators are always very sparse (*58*). The corresponding connectivity graphs between spin states often contain disconnected subgraphs that correspond to non-interacting subspaces (*46*) induced by unobvious or accidental symmetries and conservation laws. They may be simulated separately.
4) Destination state screening: Detection states in magnetic resonance are very simple—typically some form of transverse magnetization. It is therefore advantageous to perform a part of the simulation in reverse—by starting from the detection state (*44*), going backwards in time, and meeting the evolving initial condition in the middle. This is possible even with dissipative dynamics (*45*) and particularly efficient for 3D experiments.

We will not touch on the technical matters of implementing all of the above, noting only that this was done (*24*) and the details are set out in the papers cited above. Fully quantum mechanical simulations of protein-size spin systems became possible as a result (*45*). Sophisticated solvers that only include Bloch equation for the spin degrees of freedom are also available (*59*–*61*).

### Optimal time stepping

Consider the task of propagating a state vector **ρ**(0) for a time *T* > 0 under a sparse and possibly dissipative time-independent evolution generator **L** of dimension *N*, where the final state vector **ρ**(*T*) is required with machine-precision accuracy on both phases and amplitudes. The solution

ρ(T)=exp(−iLT)ρ(0) (35)

has significant efficiency caveats in situations when ‖−*i***L***T*‖ ≫ 1. In the treatment below, the following common magnetic resonance settings shall be assumed:

1) The dimension of **L** is too large for diagonalization or inversion. This, and the presence of dissipative terms, rules out Padé, Chebyshev, and Newton exponentiation methods, even in cases where they could be superior to the scaled and squared Taylor series (*62*, *63*).
2) The cost of a matrix-matrix product is *N*^α^ multiplications, where α ≤ 3.
3) The cost of a matrix-vector product is *N*^β^ multiplications, where β ≤ 2.
4) The Taylor series procedure with scaling and squaring (*62*) is used to compute the matrix exponential; the scaling is done using Hager’s estimate (*32*) of the 1-norm.

With these settings in place, Eq. 35 transforms into

$$\begin{array}{c} \rho (T)={\left[\text{exp}(-iL\Delta t)\right]}^{2^{n}}\rho (0),\;\Delta t=T/2^{n},\\ n=\text{ceil}[{\text{log}}_{2}(T{\left\|L\right\|}_{1})] \end{array}$$ (36)

where *n* is the number of propagator squaring operations that is dictated by the numerical accuracy condition for the Taylor series—that all eigenvalues of −*i***L**Δ*t* be scaled into the unit circle to make sure that there is no hump (*62*). Estimates of the largest eigenvalue are expensive, but the more easily estimated 1-norm (*32*) is an upper bound.

The optimization problem is then created by the following:

1) A matrix-vector product is cheaper than a matrix-matrix product. Some of the exponentials in Eq. 36 may be sequentially multiplied into **ρ**(0) rather than squared.
2) Propagator squaring increases the time step exponentially, whereas propagator-vector products only take the system forward at a rate that is linear with respect to the number of such products.

An optimal point must therefore exist between the number of propagator squaring operations and the number of propagator-vector products; the cost functional is the total number of multiplications.

The cost of 1-norm estimation and scaling is 5*N*^β^ multiplications (*32*). The cost of computing the Taylor series indexed by *j* and involving a matrix with eigenvalues scaled inside the unit circle is determined by the upper bound on the magnitude of the last term, which is 1/*j*!, meaning that

$$j_{\text{max}}=\text{ceil}\{\Gamma^{-1}(1/\varepsilon )-1\}$$ (37)

where ε is machine precision, Γ(*x*) is Euler’s gamma function and *j*_max_ ≈ 18 for double precision arithmetic. The number of matrix-matrix products in the Taylor series procedure is therefore *j*_max_, and the total cost of computing the Taylor series is *j*_max_*N*^α^ multiplications. The cost of squaring the propagator to compensate for the scaling of −*i***L**Δ*t* is *nN*^α^ multiplications.

If we decide not to do *m* propagator-squaring operations and multiply the propagator into the state vector 2*^m^* times instead, then the total cost will be

$$\Omega (m)=[j_{\text{max}}+n-m]N^{\alpha }+2^{m}N^{\beta }k+5N^{\beta }$$ (38)

multiplications, where *k* is the number of state vectors in **ρ**(0)—there may be several. The minimum of this function with respect to *m* is

$$\frac{d}{\mathrm{dm}}([j_{\text{max}}+n-m]N^{\alpha }+2^{m}N^{\beta }l)=0\;\Rightarrow \;m={\text{log}}_{2}(\frac{N^{\alpha -\beta }}{k\text{ln}2})$$ (39)

It does not depend on the norm of the Liouvillian, the cost of its calculation, or machine precision. While α and β strongly depend on the sparsity of the matrix, their difference does not: α − β ≈ 1. Imposing the non-negative integer constraint produces the optimal number of squaring operations to skip

$$m_{\text{opt}}=\text{max}\{\begin{array}{cc} 0 & \text{ceil}[{\text{log}}_{2}(N^{\alpha -\beta }/k\text{ln}2)] \end{array}\}$$ (40)

where ceiling is preferred to the floor because sparse matrices fill up when multiplied, and each subsequent propagator squaring will be more expensive than the previous one. Equation 40 is used in *Spinach* (*24*) whenever the explicit matrix representation of the evolution generator is available.

## PRACTICAL EXAMPLES

That MRI eventually starts to explore sophisticated multispin effects is inevitable; in some areas, it is happening already (*10*, *16*, *18*, *19*, *64*–*66*). This section is a small collection of relevant simulations illustrating scenarios where the presence of spatial dynamics makes it impractical to simulate the corresponding experiment pixel by pixel—and even that would of course have been an instance of a Kronecker product. All illustrations come from the *Spinach* 2.4 example set.

### DPFGSE excitation and suppression

A simple but important spatially encoded magnetic resonance experiment is DPFGSE (double pulsed field gradient spin echo) signal suppression, a special case of the excitation sculpting technique (*67*). It is used in the NMR spectroscopy of proteins and in localized magnetic resonance spectroscopy (MRS) of metabolites, where the 110 M signal of water protons is suppressed to the level that permits acquisition of high-quality spectra of millimolar concentrations of other molecules (*68*). Only one gradient axis (and therefore one spatial dimension) is required (Fig. 3)—this is the simplest example of the formalism presented above.

Table 2 illustrates the problem previously faced in such simulations: The Liouville space dimension is in the thousands, and accurate digitization of the gradient spirals in this case requires at least 500 points in the spatial grid. There are 4096 spin states in the Liouville space of GABA and 16 for water. Even after unimportant and unpopulated states are dropped (*45*), this still leaves us with 1912-dimensional spin operator matrices. In principle, simulating 500 independent voxels under these conditions is not difficult. The problems begin when diffusion and flow must be considered because they couple the voxels and force the direct product treatment. Matrix dimension exceeds 2 million and even the sparse representation of the evolution generator is in the hundreds of megabytes. Explicit calculation of the exponential propagator becomes impractical because the memory requirements go into multiple gigabytes. At the same time, the polyadic representation of the evolution generator takes up… 640 kB, the exact amount that Bill Gates had claimed “ought to be enough for anybody” in the year that IK was born.

**Table 2:** Matrix dimension and memory footprint statistics for the simulation of DPFGSE water suppression and selective excitation NMR spectrum shown in the bottom row of Fig. 4. Memory utilization is quoted as reported by *Matlab* using compressed column sparse format (*74*).

**Problem parameter**	**Value**	**Notes**
Liouville space dimension, full	4,112	4^6^ (GABA) + 16 (water)
Liouville space dimension, reduced	1,912	IK-2 basis (*45*)
Min. points in the spatial grid	500	Spatial Nyquist condition
Space(x)Spin dimension, full	2,056,000	500 × (4^6^ + 16)
Space(x)Spin dimension, reduced	195,500	IK-2 basis (*45*), ZTE (*42*)
Nonzeroes in evolution generator, matrix	18,252,000	**446 MB**
Nonzeroes in evolution propagator, matrix	>10^9^	**>16 GB**
Nonzeroes in evolution generator, polyadic	28,418	**640 kB**

**Fig. 4:**
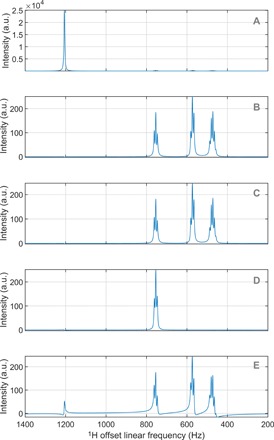
Examples of DPFGSE simulations. (**A**) Simulated ^1^H NMR spectrum of 20 mM GABA (a common metabolite) dissolved in water; water signal dominates the spectrum. (**B**) The same spectrum simulated in the presence of double pulsed field gradient spin echo (DPFGSE) water suppression. Amplitudes of first and second DPFGSE gradients are 0.1 and 0.15 T/m, respectively; gradient durations are all 1.0 ms; sample size is 15 mm; there are 500 points in the spatial grid; off-resonance selective inversion pulses at the frequency of the water signal are simulated with the Fokker-Planck method (*51*) and use 10-point Gaussian envelopes. (**C**) Same as (B), but including spatial diffusion with a diffusion coefficient of 2.6 × 10^−9^ m^2^/s. (**D**) Same as (C), but using DPFGSE signal selection rather than suppression mode. One of the GABA multiplets is selectively excited. (**E**) Same as (C), but also including spatial flow at 10 mm/s. All simulations run in minutes on a Tesla K40 graphics processing unit (GPU) using *Spinach* 2.4 and later, and are included in the example set.

A relevant point is that neither the water signal nor the signal being selectively excited is on resonance in Fig. 4. The shaped inversion pulse is simulated as an off-resonance soft pulse using an additional degree of freedom—the RF phase

$$\begin{array}{c} \frac{\partial }{\partial t}\rho (\varphi ,r,t)=[-iH(\varphi ,r,t)+R(r)+\omega_{\text{RF}}(t)\frac{\partial }{\partial \varphi }]\rho (\varphi ,r,t)\\ H(\varphi ,r,t)=H_{0}(r,t)+a(r,t)[S_{X}\text{cos}(\varphi +\varphi_{0})+S_{Y}\text{sin}(\varphi +\varphi_{0})] \end{array}$$ (41)

Here, we effectively have a “flow” forward along the RF phase coordinate φ with a velocity ω_RF_(*t*) and a periodic boundary condition. This is advantageous: Frequency-amplitude representations of NMR pulses are simpler and easier to digitize than Cartesian representations—chirp pulses are a good example (*51*). Treating RF phase as an extra spatial coordinate adds another Kronecker product to the chain, and it is handled in the same way; the technical details are published elsewhere (*51*). The salient point is that the ∂/∂φ operator responsible for turning the RF phase in Eq. 41 is time independent. Only if the frequency moves during the pulse does this term need to be taken out of the background evolution generator and sliced up. This is the other advantage of treating spin and classical dynamics at the same level: The classical coordinates need not be Cartesian.

### Point resolved spectroscopy

Point resolved spectroscopy (PRESS) relies on selective excitation of a specific volume within the MRI sample (*5*). Such excitation is straightforward—essentially three slice selection events in three orthogonal directions, followed by the rest of the desired NMR pulse sequence. However, the simulation of such an experiment involves spatially distributed multispin systems that potentially exhibit diffusion and flow—precisely the setting that this paper seeks to cover.

**Fig. 5:**
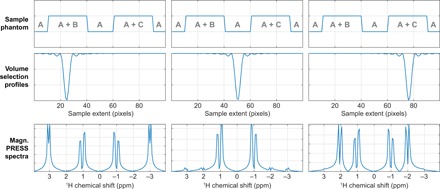
PRESS simulations for a 1D sample containing varying concentrations of three different two-spin systems. (Top) Concentration profiles of the three spin systems—system A, δ = ±1 ppm, *J* = 30 Hz; system B, δ = ±3 ppm, *J* = 10 Hz; system C, δ = ±2 ppm, *J* = 20 Hz. (Middle) Volume selection profiles excited by an off-resonance square pulse at three different frequencies. (Bottom) Magnitude mode PRESS NMR spectra of the three selected volumes. All simulations run in seconds on a Tesla K40 GPU using *Spinach* 2.4 and later, and are included in the example set.

**Fig. 6:**
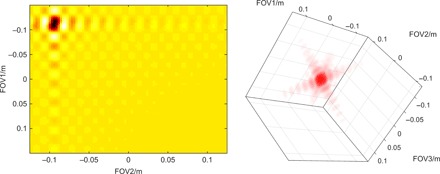
Simulation of PRESS excitation hotspots. The sample on the left has two dimensions (108 × 90 pixels), and the sample on the right has three (108 × 90 × 111 pixels). PRESS hotspots in two (108 × 90 pixels, left) and three (108 × 90 × 111 pixels, right) dimensions. All parameters and *J*-coupled spin systems are the same as in Fig. 5. Although its effect is not immediately visible, the 3D simulation includes isotropic diffusion with *D* = 2.6 × 10^−9^ m^2^/s to emphasize the ability of the formalism presented here to handle spatial dynamics in 3D. Gradients are tilted relative to the spatial grid by arbitrary angles. Both simulations (including volumetric visualization in the right panel) run in minutes to hours on a Tesla K40 GPU using *Spinach* 2.4 and later, and are included in the example set.

1D and 2D phantoms (Fig. 5 and the left panel of Fig. 6) are straightforward—careful control of matrix sparsity and Krylov propagation keeps matrix dimensions at a manageable level of several hundred thousand in the 1D case and several million in the 2D case. The situation changes radically for the 3D simulation (Table 3 and the right panel of Fig. 6) particularly when diffusion is present: Not only is the overall dimension close to being unmanageable, the presence of diffusion also introduces many nonzeroes. The result is that the size of the evolution generator is in the gigabytes (Table 3), and its exponential propagator cannot be computed.

**Table 3:** Matrix dimension and memory footprint statistics for the simulation of 3D PRESS excitation profile in the right panel of Fig. 6. Memory utilization is quoted as reported by *Matlab* using compressed column sparse format (*74*).

**Problem parameter**	**Value**	**Notes**
Liouville space dimension, full	4096	4^6^
Liouville space dimension, reduced	48	3 × 4^2^
Spatial grid, points	108 × 90 × 111	
Space(x)spin dimension, full	4.4 × 10^9^	4^6^ × 108 × 90 × 111
Space(x)spin dimension, reduced	5.2 × 10^6^	(3 × 4^2^) × 108 × 90 × 111
Nonzeroes in evolution generator, matrix	425,094,480	**11 GB**
Nonzeroes in evolution generator, polyadic	5,403,402	**233 MB**

However, the polyadic representation has no difficulty with this simulation—the evolution generator takes up 233 MB of memory. The number of nonzeroes in the polyadic representation will likely reduce further as the implementation is optimized: Unit matrices may not only be skipped at the multiplication stages but also not be stored to begin with—only their dimension is in practice needed.

### Ultrafast and pure-shift NMR spectroscopy

**Fig. 7:**
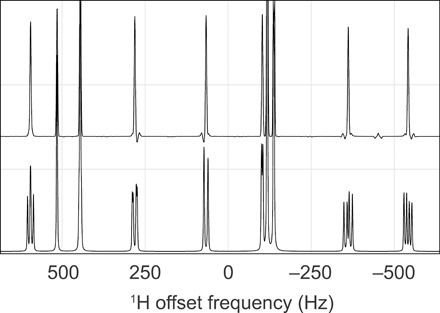
PSYCHE (pure shift yielded by chirp excitation) strong coupling artefact simulation. The figure is zoomed into the central parts of the simulations of 500 MHz NMR (bottom) and PSYCHE (*69*) (top) spectra of rotenone (22 proton spins). The simulation runs in minutes on a Tesla K40 GPU using *Spinach* 2.4 and later. The standard NMR simulation is nearly instantaneous.

Ultrafast (*3*) and spatially encoded pure-shift (*69*) NMR spectroscopy use spatial encoding to replace what was originally an extra temporal dimension in the earlier NMR pulse sequences. Both subjects are vast, but the conclusions from the simulations of the corresponding experiments (Fig. 7) are exactly the same as above—polyadic representation of the evolution generators either makes the simulation possible to start with or reduces the memory requirements by orders of magnitude relative to the sparse matrix representation of the corresponding operators. The reduction is particularly noticeable when spatial dynamics is present. Artifacts in ultrafast and pure-shift spectroscopy can be dominated by spatial dynamics effects—the ability to simulate those will be a welcome development.

Because we focus here specifically on spatially distributed multispin processes, it is useful to find a spatially encoded magnetic resonance experiment that uses the relevant properties to the maximum possible extent. Such an experiment appeared very recently—the ultrafast “maximum-quantum” sequence (*70*) uses spatial encoding to create a 2D correlation spectrum between the chemical shifts of the standard transverse magnetization and the chemical shift of the highest coherence order achievable in the system. Such experiments are impossible to simulate unless detailed quantum mechanical treatment is performed in the spin subspace. The spectra themselves are nothing special—a few peaks on the 2D plane that tell something useful to chemists (*70*)—but wall clock times and matrix dimension statistics are again pertinent (Table 4).

**Table 4:** Evolution generator dimension (spin × spatial), number of nonzeroes (nnz), and matrix-vector multiplication wall clock time (wct) statistics for a series of simulations of ultrafast “maximum-quantum” experiments (*70*) that include the effect of isotropic spatial diffusion using seven-point central finite difference operators.

**System**	**Parameter**	**Value**	**Notes**
2-Spin	State space dimension	16 × 500	Sparse matrix representation fits into the L2 cache—polyadic processing is slower, memory footprint improvement is not significant
	nnz(**L**) and wct(**Lv**)^a^, sparse	116,000, 0.5 ms	
	nnz(**L**) and wct(**Lv**)^a^, polyadic	12,000, 5 ms	
4-Spin	State space dimension	256 × 500	Sparse matrix representation exceeds L2 cache, parity on the wall clock time, significant memory advantage for the polyadic format
	nnz(**L**) and wct(**Lv**)^a^, sparse	2,600,000, 13 ms	
	nnz(**L**) and wct(**Lv**)^a^, polyadic	14,000, 18 ms	
6-Spin	State space dimension	4096 × 500	Sparse matrix representation is in the gigabytes, polyadic processing is faster and offers a vast memory footprint advantage
	nnz(**L**) and wct(**Lv**)^a^, sparse	37,000,000, 227 ms	
	nnz(**L**) and wct(**Lv**)^a^, polyadic	87,000, 185 ms	

^a^Intel Xeon E5-2698, single core.

In common with the rest of the simulation methods that went into *Spinach*, polyadic generator storage does not offer any advantages for small spin systems on small grids (Table 4, two-spin system). The advantage is in the scaling—the situation changes radically as the system gets larger: Wall clock time parity is achieved with a four-spin system, and a wall clock advantage appears thereafter. At the same time, the improvement in the memory footprint is dramatic—what is supposed to be a 1,228,800-dimensional matrix only has 87,400 nonzeroes in the polyadic representation, and at least half of that comes from unit matrix operands whose explicit storage is not strictly required. For the six-spin system, the number of nonzeroes in the polyadic representation of the evolution generator is much smaller than the number of nonzeroes in the state vector.

## CONCLUSIONS AND OUTLOOK

Spatially encoded magnetic resonance spectroscopy of large spin systems distributed and moving in three spatial dimensions can now be simulated in reasonable time and essentially without approximations. This work describes the general structure of the mathematical methods and the software engineering involved. The key design decision was to avoid opening certain Kronecker products in the algebraic structure of the evolution generator. An open-source software implementation is already available (*24*); the immediate applications have been to ultrafast (*71*), spatially encoded pure-shift (*72*), and diffusion (*73*) NMR spectroscopy where spatial dynamics is inextricably linked to multispin processes. As the MRI community starts making greater use of multispin effects and metabolites in their pulse sequences (*10*, *16*, *18*, *19*, *64*–*66*), we expect these simulation tools to find further applications in MRI experiment design.
